# Rural-urban disparities in preventive breast and cervical cancer screening among women with early-onset dementia

**DOI:** 10.1186/s12905-023-02301-7

**Published:** 2023-05-11

**Authors:** Wendy Y. Xu, Eli Raver, Jeah Jung, Yiting Li, Gaby Thai, Sunmin Lee

**Affiliations:** 1 Division of Health Services Management and Policy, College of Public Health, The Ohio State University, 1841 Neil Ave., 200-D Cunz Hall, Columbus, OH 43210 USA; 2 Department of Health Administration and Policy, College of Public Health, George Mason University, Fairfax, USA; 3 Department of Neurology, School of Medicine, University of California, Irvine, USA; 4 Department of Medicine, School of Medicine & Chao Family Comprehensive Cancer Center, University of California, Irvine, USA

**Keywords:** Preventive cancer screening, Breast cancer, Cervical cancer, Alzheimer’s disease and related dementias

## Abstract

**Background:**

The early onset of Alzheimer’s disease and related dementias (ADRD) before age 65 can introduce life and health care complications. Preserving an early-onset ADRD patient’s daily functioning longer and delaying declines in health from non-ADRD conditions become important preventive goals. This study examined the differences in utilization of preventive cancer screenings between patients with and without early-onset ADRD, and compared utilization of the screenings in rural versus urban areas among women with early-onset ADRD in the United States.

**Methods:**

We conducted a cross-sectional study of women aged 40 to 64 years eligible for mammogram and cervical cancer screenings using commercial insurance claims from 2012 to 2018. We measured the use of biennial mammogram among women 50 to 64 years old, and the use of triennial Pap smear test among women 40 to 64 years old. We used inverse probability weighted logistic regressions to estimate the odds of receiving preventive cancer screenings by the presence of early-onset ADRD or cognitive impairments (CI). We used multivariable logistic regressions to estimate the odds of receiving preventive cancer screenings by rural or urban residence among women with early-onset ADRD/CI.

**Results:**

Among 6,349,308 women in the breast cancer screening sample (mean [SD] age, 56.52 [4.03] years), 36,131 had early-onset ADRD/CI (mean [SD] age, 57.99 [3.98] years). Among 6,583,088 women in the cervical cancer screening sample (mean [SD] age, 52.37 [6.81] years), 30,919 had early-onset ADRD/CI (mean [SD] age, 55.79 [6.22] years). Having early-onset ADRD/CI was associated with lower utilization of mammogram (OR: 0.92, 95% CI: 0.90–0.95). No significant difference was observed in Pap smear screening (OR: 0.99, 95% CI: 0.96–1.02) between patients with and without early-onset ADRD/CI. Among patients with early-onset ADRD/CI, those in rural areas were less likely than those in urban areas to have mammograms (OR: 0.91, 95% CI: 0.85–0.97) and Pap smears (OR: 0.65, 95% CI: 0.61–0.71).

**Conclusions:**

The observed pattern of rural-urban differences in cancer screening in our study emphasizes the need for efforts to promote evidence-based, individualized decision-making processes in the early-onset ADRD population.

## Introduction

Alzheimer’s disease and related dementias (ADRD) are irreversible and debilitating conditions that cause loss of memory, cognition, and independent functioning. While most of the ADRD diagnosis happens in “old age”, nearly 6% of patients with ADRD have an early onset before age 65. ADRD have affected a growing number of younger patients, and it is estimated that 220,000 to 640,000 Americans live with early-onset ADRD in the U.S [1]. Most patients with early-onset ADRD are still in the workforce and may even be caregivers to their family members when they first develop dementia. On average, patients live for around eight to 10 years after the diagnosis of dementia [2]. Therefore, preserving one’s daily functioning longer and delaying declines in health from non-ADRD conditions, such as breast and cervical cancers, can be important preventive care goals for patients with early-onset ADRD.

The United States Preventive Services Task Force (USPSTF) guidelines recommend mammogram screening for women 50–74 years of age and cervical cancer screening to women 30–65 years of age. The majority of breast and cervical cancer cases in the U.S. develop in women less than 65 years old [3–5]. While the early-onset population is growing, there is scant literature on cancer screening use among these younger patients with ADRD.

Breast and cervical cancer screenings allow detection of cancer at earlier stages to significantly reduce the incidence of advanced cancers. Earlier cancer detection can allow time for patients with early-onset ADRD and family members to discuss preferences for treatments and to process advanced care planning. Moreover, family members may want early-onset patients to still receive breast and cervical cancer screenings. For example, if there exist hereditary risks of cancers among female family members (mothers, daughters, and sisters), assessment of cancer risks and early detection of those risks would be invaluable for care planning.

While USPSTF recommends these evidence-based screenings, complexities related to decision/use of screening tests arise with comorbid conditions. The presence of ADRD makes it very challenging to deliver health care. ADRD patients may rely on caregivers to navigate the healthcare system. In addition, the cognitive or behavioral symptoms of dementia may generate additional burdens to screening. For example, though considered non-invasive, mammogram and Pap tests require nudity and introduce discomfort to patients, particularly to those with cognitive impairments. Because of this complexity, qualitative clinical research has found that caregivers’ views toward cancer screening for ADRD patients varied substantially. Caregivers of women with mild and moderate dementia valued mammogram and planned to continue, while caregivers of those with severe dementia did not consider cancer screening highly important [6]. This suggests that patients with early-onset ADRD may have different care goals and present different patterns of preventive screening use than the general population.

Conventionally, access to preventive screenings has been limited in rural areas due to provider scarcity and long travel distances [7–9]. This further complicates the cancer screenings by rural early-onset patients because these areas have fewer clinicians who would be able to help balance the benefits of cancer screening and the associated risks while coordinating individualized decisions. Therefore, patients with early-onset ADRD may be particularly vulnerable to the intersection of rural health care disparity and the presence of ADRD.

There is growing research to address the important question of whether continued preventive screenings for geriatric dementia patients is appropriate. This literature often recommends shared decision-making tailored to individuals’ needs when it comes to preventive screening use [10, 11]. However, little attention is paid to producing information about screening decision-making for younger women with early-onset dementia, especially those who are still covered by employer-sponsored insurance plans. These women or their spouses are still working and most have children in the family. Little knowledge exists about the current state of screenings in early-onset women with ADRDs and about key factors (e.g., rurality) that may affect use of preventive screening tests in this population. This knowledge gap hampers further research that would help develop individualized interventions. Thus, our study aims to (1) compare the use of biennial mammogram and triennial Pap smear screenings between women with early-onset ADRD and those without, and (2) compare the use of mammogram and Pap smear screenings between women in rural and urban areas among those with early-onset ADRD. The burden of ADRD on the community and the health care system is expected to increase in the next two decades as the number of patients with ADRD continues to grow [12]. Examining the level of utilization of preventive cancer care and identifying the role of rurality in cancer screening use among patients with early-onset ADRD is the first step toward promoting individualized screening decision making and identifying target patient groups for whom preventive services can be beneficial.

## Methods

### Data

We conducted a retrospective cross-sectional study using the 2012–2018 IBM-Watson MarketScan Commercial Claims and Encounters Database [13]. This nationwide administrative claim database includes detailed information regarding treatment episodes, such as detailed diagnoses, procedures, and care settings for commercially insured individuals. The data also include enrollee demographics, such as age, sex, and rural residency, and health plan information including monthly insurance enrollment status and insurance plan types. The Ohio State University Institutional Review Board (IRB) approved this study (Federal wide Assurance #00006378; IRB approval #2021B0161).

### Preventive cancer screening measures

We constructed preventive cancer screening measures based on the age and frequency guidelines from the USPSTF levels A or B recommendations. We adopted guidelines that were in effect during our study period. The USPSTF guideline between 2009 and 2016 recommended biennial mammography for women aged 50 to 74 years to screen for breast cancer [14]. The USPSTF guideline between 2012 and 2018 recommended Pap smear alone every 3 years or Pap smear with human papillomavirus (HPV) testing every 5 years for women aged 30 to 65 to screen for cervical cancer [15]. Thus, our study measured biennial mammogram among women 50–64 during a 2-year continuously enrolled period, and triennial Pap smear test among women 40–64 during a 3-year continuously enrolled period (women were considered in compliance if they had a co-testing within 5 years when data was available).

Claims-based definitions from the Healthcare Effectiveness Data and Information Set (HEDIS) algorithms were used to identify cancer screenings in the outpatient claims data using the Current Procedure Terminology codes and International Classification of Diseases 9th or 10th revision (ICD-9 or ICD-10) diagnosis codes [16]. Screenings furnished due to surveillance or diagnostic purposes were excluded to capture preventive screenings, based on the HEDIS algorithms.

### Study sample

The study included two samples based on the age range required for the screening in question: 50-64-year-old women for the mammogram sample; and 40-64-year-old women for the Pap smear sample. We required individuals to be continuously enrolled in employer-sponsored health insurance for the full calendar year with medical coverage. This enrollment requirement helped us capture the most complete information on health services utilization.

### Identifying patients with early-onset ADRD

We followed the algorithm from the Centers for Medicare & Medicaid Services (CMS) and ICD-9 or ICD-10 codes to identify early-onset ADRD patients in the population of the age range specified above. We identified patients with any ADRD diagnosis in outpatient or inpatient claims, in any year during the observation period [17]. The conditions of cognitive impairment (CI) are often related to dementia, and CI is an intermediate state between normal cognition and formal dementia diagnosis. Therefore, we also identified patients with cognitive impairment (CI) using ICD-10 code G31.84 or ICD-9 code 331.83 based on neurologists’ coding practice.

### Statistical analyses

We performed two analyses. We first compared cancer screening utilization between women with and without early-onset ADRD/CI. The key explanatory variable in this analysis was having early-onset ADRD or CI. Logistic regression was used to estimate the odds ratio of using a recommended cancer screening between women with and without early-onset ADRD/CI, adjusting for individual-level variables associated with health care use. We used inverse probability treatment weighting (IPTW) to balance observed characteristics between patients with and without early-onset ADRD/CI exposure. Robust standard errors clustered by individual enrollees accounted for the fact that individuals could be observed multiple times during the study interval. All p-values were from 2-sided tests and results were considered statistically significant at p < .05. Stata, version 14 (StataCorp) was used for statistical analysis.

The control variables included age (represented by age groups: 40–44 (reference for Pap smear sample), 45–49, 50–54 (reference for mammogram sample), 55–59, and 60–64), rurality, and enrollee plan characteristics as reflected by plan types. Consistent with prior literature, [18] rural status was measured as a binary variable based on metropolitan statistical areas (MSAs) defined by the US Office of Management and Budget. The rural area was a non-MSA area. We measured plan types based on out-of-pocket expectations (e.g. deductibles), whether the plan focuses on care management to reduce health care costs (e.g. health maintenance organization (HMO), Preferred Provider Organization (PPO)), and some health care provider features built into different plan types (e.g., the need for referrals to specialists). Charlson Comorbidity Index scores were used to measure other chronic condition burdens using ICD-9/ICD-10 diagnosis codes. The conditions of dementia were excluded from this calculation because it was separately measured as a variable of interest.

The second analysis compared use of preventive cancer screenings between women in rural and urban areas among those with ADRD/CI. The key explanatory variable for this analysis was rurality. We estimated logistic regressions to model the likelihood of screening utilization based on individual characteristics as described above, limiting the sample to women with early-onset ADRD/CI. We estimated odds ratios of having recommended screening services based on rural residency controlling for age, comorbidity, and health plan type, in the early-onset ADRD population. Based on these logistic regression models, we calculated the adjusted rates of cancer screening utilization for women with early-onset ADRD/CI in rural and urban areas.

## Results

Table 1 shows descriptive statistics of each study sample. The mammogram sample included 16,644,386 person-year observations for 6,349,308 women. On average, 14.6% of the mammogram sample were in rural areas. The most common health plan type was PPO plans, covering 59.4% of women. The average utilization rate of biennial mammogram was 68.5%.

**Table 1:** Characteristics of Study Samples

	Mammogram Sample			Pap Smear Sample		
	Total	ADRD/CI	non-ADRD/CI	Total	ADRD/CI	non-ADRD/CI
**Preventive Cancer Screening Compliance**, %	68.50	63.97	68.51	62.72	55.41	62.74
**Having ADRD/CI**, %	0.28	100	0	0.23	100	0
**Rural**, %	14.61	13.61	14.62	13.48	12.97	13.48
**Age**, mean (SD), years	56.52 (4.03)	57.99 (3.98)	56.51 (4.03)	52.37 (6.81)	55.79 (6.22)	52.36 (6.81)
**Age 40–44**, %	N/A	N/A	N/A	16.92	6.97	16.94
**Age 45–49**, %	N/A	N/A	N/A	19.02	10.81	19.04
**Age 50–54**, %	35.54	22.79	35.58	21.86	18.23	21.87
**Age 55–59**, %	36.47	34.63	36.47	23.43	28.37	23.42
**Age 60–64**, %	27.99	42.58	27.95	18.77	35.63	18.73
**Charlson Comorbidity Index**, mean (SD)	0.57 (1.21)	1.64 (2.24)	0.57 (1.20)	0.49 (1.12)	1.60 (2.23)	0.49 (1.12)
**Insurance plan type**, %	--	--	--	--	--	--
Comprehensive	4.12	6.81	4.11	3.47	6.48	3.46
HMO	11.22	10.29	11.22	10.97	9.50	10.98
PPO	59.41	58.61	59.41	57.50	57.43	57.50
POS	7.95	8.43	7.95	7.61	8.42	7.61
HDHP/CDHP	16.15	14.76	16.16	19.44	17.16	19.44
EPO	1.15	1.10	1.15	1.01	1.01	1.01
**Unique Individuals, n** ^**a**^	6,349,308	36,131	6,340,838	6,583,088	30,919	6,575,926
**Observations, n, person-years** ^**b**^	16,644,386	46,246	16,598,140	16,605,918	38,704	16,567,214

Abbreviations: ADRD, Alzheimer’s disease and related dementias; CI, cognitive impairment; EPO, exclusive provider organization; HMO, health maintenance organization; POS, point of service; PPO, preferred provider organization; HDHP, high deductible health plan; CDHP, consumer directed health plan

^a^ The sum of unique individuals in each group (ADRD/CI and non-ADRD/CI) is greater than the sample total because some individuals developed ADRD/CI during the study period

^b^ Person-year observations are the denominator for descriptive statistics

The Pap smear sample included 16,605,918 person-year observations for 6,583,088 women. In this sample, 13.5% of women were in rural areas and the most common health plan type was PPO (57.5%). The average rate of receiving recommended Pap smear (triennial or every 5 years with HPV co-testing) was 62.7%.

Table 2 shows the results of the IPTW logistic regression models, accounting for individual characteristics in the modeling. Having early-onset ADRD/CI was associated with lower utilization of recommended mammograms compared to women without early-onset ADRD/CI (OR: 0.92, 95% CI: 0.90–0.95). However, there was no significant difference in Pap smear rates between women with and without early-onset ADRD/CI (OR: 0.99, 95% CI: 0.96–1.02).

**Table 2:** Inverse Probability Treatment Weighted Regression Results Estimating Cancer Screening Utilization between Women with and without Early-onset ADRD/CI

	Mammogram, Odds ratio (95% CI)	Pap Smear, Odds ratio (95% CI)
ADRD/CI	0.92 (0.90, 0.95)	0.99 (0.96, 1.02)
Rural	0.90 (0.87, 0.94)	0.67 (0.65, 0.70)
Age 40–44	N/A	1 [Reference]
Age 45–49	N/A	0.84 (0.79, 0.89)
Age 50–54	1 [Reference]	0.67 (0.63, 0.71)
Age 55–59	0.99 (0.96, 1.02)	0.51 (0.48, 0.54)
Age 60–64	0.98 (0.95, 1.01)	0.41 (0.38, 0.43)
Charlson Comorbidity Index	0.99 (0.98, 1.00)	0.94 (0.93, 0.94)
Comprehensive Health Plan	1 [Reference]	1 [Reference]
EPO	1.44 (1.26, 1.64)	1.58 (1.37, 1.82)
HMO	2.13 (2.00, 2.27)	1.45 (1.35, 1.56)
POS	1.82 (1.70, 1.94)	1.57 (1.45, 1.69)
PPO	1.67 (1.59, 1.76)	1.47 (1.38, 1.55)
HDHP/CDHP	1.87 (1.76, 1.98)	1.63 (1.53, 1.74)
(Intercept)	1.31 (1.24, 1.38)	1.96 (1.83, 2.11)

Abbreviations: ADRD, Alzheimer’s disease and related dementias; CI, cognitive impairment; EPO, exclusive provider organization; HMO, health maintenance organization; POS, point of service; PPO, preferred provider organization; HDHP, high deductible health plan; CDHP, consumer directed health plan

Table 3 shows the results of the logistic regression models comparing cancer screening utilization between women with early-onset ADRD/CI in rural areas and those in urban areas, controlling for individual characteristics. Women with early-onset ADRD/CI in rural areas were significantly less likely than those in urban areas to have recommended mammograms (OR: 0.91, 95% CI: 0.85–0.97). Women with early-onset ADRD/CI in rural areas were much less likely than those in urban areas to have recommended Pap smears—the early-onset ADRD patients in rural areas saw 35% lower odds of adhering to cervical cancer screening (OR: 0.65, 95% CI: 0.61–0.71).

**Table 3:** Logistic Regression Results Estimating Rurality and Cancer Screening Utilization among Women with Early-onset ADRD/CI

	Mammogram, Odds ratio (95% CI)	Pap Smear, Odds ratio (95% CI)
Rural residence	0.91 (0.85, 0.97)	0.65 (0.61, 0.71)
Age 40–44	N/A	1 [Reference]
Age 45–49	N/A	0.80 (0.71, 0.90)
Age 50–54	1 [Reference]	0.62 (0.55, 0.69)
Age 55–59	0.93 (0.88, 0.99)	0.46 (0.41, 0.51)
Age 60–64	0.86 (0.81, 0.91)	0.35 (0.32, 0.39)
Charlson Comorbidity Index	0.92 (0.91, 0.93)	0.91 (0.90, 0.92)
Comprehensive Health Plan	1 [Reference]	1 [Reference]
EPO	1.78 (1.40, 2.25)	2.03 (1.56, 2.63)
HMO	2.48 (2.21, 2.77)	1.80 (1.58, 2.05)
POS	2.15 (1.91, 2.41)	1.78 (1.56, 2.04)
PPO	2.00 (1.82, 2.19)	1.67 (1.50, 1.86)
HDHP/CDHP	2.25 (2.03, 2.50)	1.91 (1.70, 2.16)
(Intercept)	1.14 (1.03, 1.26)	1.84 (1.60, 2.13)

Abbreviations: ADRD, Alzheimer’s disease and related dementias; CI, cognitive impairment; EPO, exclusive provider organization; HMO, health maintenance organization; POS, point of service; PPO, preferred provider organization; HDHP, high deductible health plan; CDHP, consumer directed health plan

**Fig. 1:**
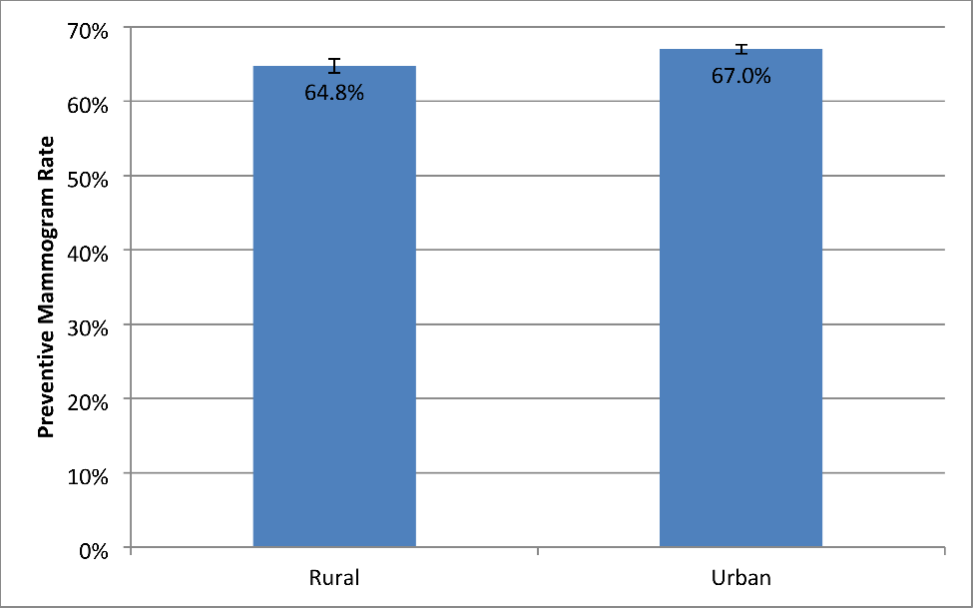
Adjusted breast cancer screening rates among rural and urban patients with early-onset ADRD/CI Abbreviations: ADRD, Alzheimer’s disease and related dementias; CI, cognitive impairment

**Fig. 2:**
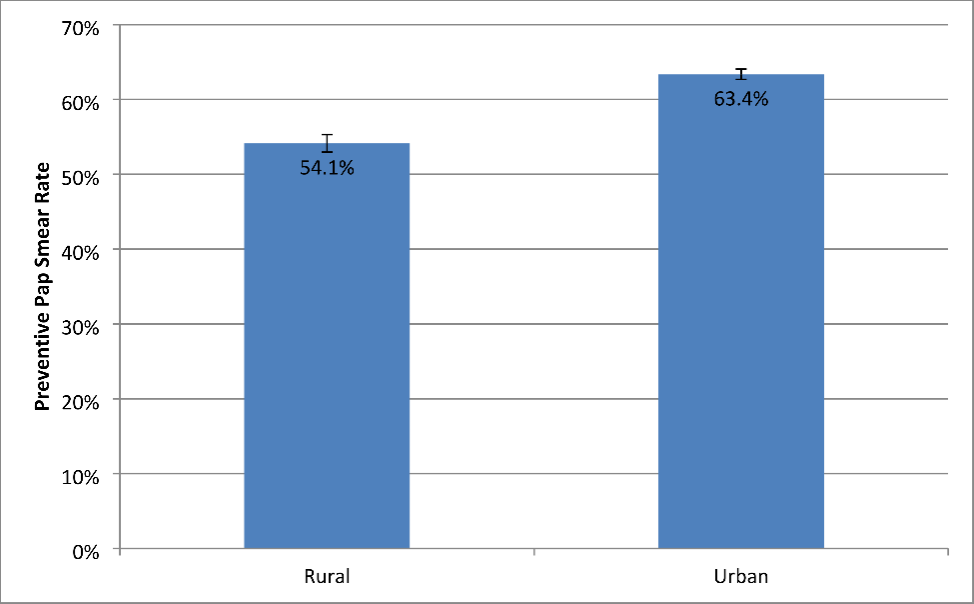
Adjusted cervical cancer screening rates among rural and urban patients with early-onset ADRD/CI Abbreviations: ADRD, Alzheimer’s disease and related dementias; CI, cognitive impairment

Figure 1 shows the adjusted breast cancer screening rates based on the logistic regression models comparing women with early-onset ADRD/CI in rural and urban areas. Adjusting for individual characteristics, the rate of mammogram use was 64.8% (95% CI: 63.8%, 65.7%) for women with early-onset ADRD/CI in rural areas, compared to 67.0% (95% CI: 66.4%, 67.6%) for women with early-onset ADRD/CI in urban areas. Figure 2 shows the adjusted cervical cancer screening rates based on the logistic regression models comparing women with early-onset ADRD/CI in rural and urban areas. The adjusted rate of Pap smear use was 54.1% (95% CI: 53.0%, 55.3%) for women with early-onset ADRD/CI in rural areas, compared to 63.4% (95% CI: 62.7%, 64.0%) for women with early-onset ADRD/CI in urban areas.

## Discussion

ADRD are growing epidemics in the U.S. Early-onset dementia patients comprise a relatively small subset, but the number of those patients is growing [19]. Our study delivers the first empirical evidence on how early-onset dementia is associated with use of preventive cancer screenings. In addition, we report information on how screening use differs between rural and urban areas among patients with early-onset ADRD. This provides the important baseline information about preventive cancer screening in younger patients with dementia. This information can be used to help advance the development of interventions promoting individualized decision making on cancer screening to best benefit younger patients with dementia.

Using national insurance claims data from commercially insured women, we found that women with early-onset ADRD/CI had lower compliance with mammogram but not Pap smear, compared with those without early-onset ADRD/CI. Early-onset ADRD/CI patients used less mammograms, likely because the cognitive impairments gradually cause a patient to lose the ability to self-manage health conditions and maintain independence, posing substantial challenges to primary and secondary care [20–23]. Our findings of Pap smear use suggest that younger patients with dementia were still engaged in cervical cancer screening, despite the invasiveness of the procedure, at a similar level to those without dementia. There are risks associated with the screening and potential downstream treatment costs and side effects. Thus, views about potential benefits of cancer screening tests are mixed [6, 24, 25]. Perceived values of screening and decisions on use of screenings are likely to vary across early-onset ADRD patients and their caregivers. This variation may have led to our different findings between mammogram and Pap smear test use.

Our analysis also highlighted the discrepancies in screening compliance in rural patients with early-onset ADRD relative to those in urban areas. Variation in the use of preventive cancer screening among patients with early onset ADRD/CI suggests that the level of screening use is not entirely driven by the complex clinical features of ADRD. The lower level of cancer screening rates in rural areas is not surprising because general health care challenges in rural settings have been documented in the literature among patients with other conditions or geriatric ADRD [8, 9, 26]. Our study contributes to the literature by reporting differences in screening use among rural and urban areas among early-onset ADRD patients. These baseline screening rates are useful to explore any future interventions of individual screening decision making.

Patients with early-onset ADRD in rural settings may present compounded challenges for several reasons. First, the existing structural barriers to accessing care in rural areas, such as shortage of providers, may contribute to the lower level of screening use in early-onset dementia patients. Family caregivers must juggle between priorities of care for early-onset patients, and long travel distances may compound the burdens of getting patients with cognitive issues to a care setting. In rural areas, there is also a dearth of clinicians who have the experience and bandwidth to provide adequate clinical advice and care to dementia patients. Therefore, the referral of preventive screenings may become a lower priority in the care amid the mixed views about the potential benefits of screening tests for dementia patients. Moreover, cancer treatment can be costly and extra burdensome to many rural families who live far away from oncology care facilities. This could have further discouraged early-onset ADRD patients in rural areas from receiving preventive cancer screenings.

The Alzheimer’s Association has highlighted the need for efforts to enhance person-centered quality care [1]. Therefore, health services for early-onset ADRD patients may focus on optimizing and extending functional years and enhancing the quality of life. Given the lack of definite clinical guidelines for cancer screening in ADRD patients and the uniqueness of early-onset ADRD, our findings call for more attention devoted to this population, especially those in rural areas. Many families of women with dementia may still value preventive cancer screenings and would like to continue. Yet compared with nonrural areas, the rural health care system is spread out across broader areas and often lacks necessary infrastructure—fewer cancer hospitals or treatment facilities and fewer laboratories or radiation therapy services. Rural patients often must travel far to reach oncology professionals, such as oncologists, radiologists, and surgeons. These challenges would make care for early-onset ADRD patients even more costly and difficult if they are diagnosed with cancers, diminishing the potential benefits to help with the quality of life in mid-age women with milder cognitive impairment.

Yet so far, initiatives to care for patients with cognitive impairment and dementia have focused primarily on clinical practices to manage dementia symptoms and long-term care for patients with advanced dementia. Clinical features of ADRD, such as cognitive issues and dependency, make it difficult to set an appropriate level of preventive services. Some may argue that current screening rates in older adults with dementia are high, and interventions to promote cessation of screening among ADRD patients may be beneficial [11]. Others perceive that cancer control may help to allow individuals living with dementia to remain in the community longer, especially for younger ADRD patients, with better quality of life. Cancer control among younger ADRD patients may thereby delay costly hospitalizations and residential care. This is particularly meaningful for rural ADRD patients and families who are not close to these facilities. Further, for early-onset ADRD patients who may still be in the workforce, preventive services that offer substantial benefits and avoid higher costs and treatment complications may be especially important to potentially alleviate disease burdens for patients and families. Diagnosing cancers earlier on also presents valuable opportunities for some early-onset ADRD patients to make informed decisions about treatments when they can. It allows time for family caregivers to plan for care needs and to better prevent or control symptoms and side effects from cancer treatments.

### Limitations

There are several limitations worth noting in this study. First, we could not use a nuanced definition for rurality because the data do not contain geographic information lower than the level of metropolitan statistical areas. Second, preventive cancer screenings are measured using claims-based algorithms. While the HEDIS measures are gold-standard algorithms for claims data, it still lacks certain clinical details such as cancer stages or symptoms. Further, the ADRD patients who received medical care that was paid entirely out-of-pocket would be missed in our study. Moreover, the CMS algorithm used to identify early-onset ADRD patients was developed primarily for the Medicare population. This algorithm includes both regular and early-onset diagnosis codes. We applied all the diagnosis codes from the CMS algorithm to our study population because providers may not always use early-onset-specific ICD-9/10 codes. Due to data limitations, studying the stage of cancer diagnosis for ADRD patients was beyond the scope of our study. Last, our study did not examine cancer screenings for those in Medicare or Medicaid because we did not have the data. Thus, the study findings have limited generalizability beyond the commercially insured population, although a majority of those who are insured under age 65 have private insurance in the U.S.

## Conclusion

Our study revealed that early-onset ADRD patients use less mammogram screening relative to those without early-onset ADRD. Early-onset ADRD patients in rural settings had lower mammogram or Pap smear screening rates compared to their urban counterparts. Cognitive and functional status of patients with early onset will likely continue to decline, negatively affecting the productivity and well-being of working-age individuals. The observed pattern of rural-urban differences in preventive cancer screening in our study emphasizes the need for efforts to promote evidence-based, individualized decision making processes in early-onset population.

## Data Availability

The raw data used in this study are proprietary data from Merative (formerly IBM Watson Health) and restrictions apply to the availability of these data. The data were used under license for the current study, and so are not publicly available. All our aggregated statistical results, even those unreported, are available to the public upon request to the corresponding author, Wendy Y. Xu.

## Abbreviations

ADRD: Alzheimer’s disease and related dementias
CI: Cognitive impairment
USPSTF: United States Preventive Services Task Force
HPV: Human papillomavirus
HEDIS: Healthcare Effectiveness Data and Information Set
CPT: Current Procedural Terminology
ICD-9: International Classification of Diseases, 9th Revision
ICD-10: International Classification of Diseases, 10th Revision
IPTW: Inverse probability treatment weighting
HMO: Health maintenance organization
PPO: Preferred provider organization
